# Nanocomposite Membranes for PEM-FCs: Effect of LDH Introduction on the Physic-Chemical Performance of Various Polymer Matrices

**DOI:** 10.3390/polym15030502

**Published:** 2023-01-18

**Authors:** Muhammad Habib Ur Rehman, Ernestino Lufrano, Cataldo Simari

**Affiliations:** 1 Department of Chemistry and Chemical Technologies, University of Calabria, 87036 Rende, Italy; 2 National Reference Centre for Electrochemical Energy Storage (GISEL)—INSTM, Via G. Giusti 9, 50121 Firenze, Italy

**Keywords:** proton exchange membranes, nanocomposite membranes, layered double hydroxides, swelling tests, PFG-NMR, proton conductivity, dynamic mechanical analysis, proton exchange membrane fuel cells

## Abstract

This is a comparative study to clarify the effect of the introduction of layered double hydroxide (LDH) into various polymer matrices. One perfluorosulfonic acid polymer, i.e., Nafion, and two polyaromatic polymers such as sulfonated polyether ether ketone (sPEEK) and sulfonated polysulfone (sPSU), were used for the preparation of nanocomposite membranes at 3 wt.% of LDH loading. Thereafter, the PEMs were characterized by X-ray diffraction (XRD) and dynamic mechanical analysis (DMA) for their microstructural and thermomechanical features, whereas water dynamics and proton conductivity were investigated by nuclear magnetic resonance (PFG and T_1_) and EIS spectroscopies, respectively. Depending on the hosting matrix, the LDHs can simply provide additional hydrophilic sites or act as physical crosslinkers. In the latter case, an impressive enhancement of both dimensional stability and electrochemical performance was observed. While pristine sPSU exhibited the lowest proton conductivity, the sPSU/LDH nanocomposite was able to compete with Nafion, yielding a conductivity of 122 mS cm^−1^ at 120 °C and 90% RH with an activation energy of only 8.7 kJ mol^−1^. The outcome must be ascribed to the mutual and beneficial interaction of the LDH nanoplatelets with the functional groups of sPSU, therefore the choice of the appropriate filler is pivotal for the preparation of highly-performing composites.

## 1. Introduction

Ever-increasing green energy demands due to environmental pollution caused by the consumption of fossil fuels at a high rate, have urged researchers to discover sustainable and eco-friendly alternatives. The adoption of fuel cells is now being considered one of the decisive steps for clean energy generation because only water and heat are the by-products resulting from electrochemical conversion reactions [1,2]. Among various types of fuel cells, proton exchange membrane fuel cells (PEM-FCs) have now emerged as a promising technology due to their quick start-up, high power density, low operating temperatures, and high commercialization possibilities [3]. Proton exchange membrane (PEM) is an essential component of PEM-FCs which allows the transfer of H^+^ from one electrode to another electrode. Perfluorinated membrane (Nafion^®^) developed by DuPont is considered a standard proton exchange membrane to date due to its chemical and thermal stability, high mechanical resistivity, and good ion conductivity [4]. All these attributes are owing to the hydrophobic/hydrophilic structure having polytetrafluoroethylene (PTFE) as a strong hydrophobic backbone and hydrophilic ether-linked side chains containing sulfonic acid (-SO_3_H) groups capable of transporting protons [5]. Despite all these advantages, Nafion^®^ membranes present some limitations i.e., ionconductivity drops rapidly in anhydrous conditions (RH < 80%) restricting the temperature to that not exceeding the boiling point of water and Nafion^®^ is an expensive material priced at about 500–700$ per m^2^ [6,7,8]. So over the last few years, many alternative polymeric materials for PEM have been proposed which include sulfonated polyether ether ketone (sPEEK) [9,10,11], sulfonated polysulfone (sPSU) [12,13], sulfonated polyether sulfone (sPES) [14,15,16], polybenzimidazole (PBI) [17,18,19] and sulfonated polystyrene (sPS) [20,21,22]. In addition, many research groups have prepared composite PEMs using organic and inorganic fillers like TiO_2_, ZrO_2_, ZrP, BPO_4_, graphene oxide (GO), and SiO_2_ to enhance the properties of proton exchange membranes [23,24,25,26,27,28]. More recently, innovative nanomaterials such as metal-organic frameworks (MOFs), halloysite nanotubes (HNTs), and MXene also gained increasing attention as promising inorganic fillers [29,30,31]. Overall, the strategy allowed for the successful achievement of noticeable improvements in terms of ionic conductivity and mechanical resistance. For instance, Yosuff et. al. prepared a high-temperature PBI membrane for anhydrous conditions and used sulfonated graphene oxide as an inorganic filler. They reported a two-times enhancement in proton conductivity at 150 °C after the addition of graphene oxide in the polymer matrix [32]. Heo et. al. also used sulfonated graphene oxide as a filler and studied the performance of sPEEK membranes for a direct methanol fuel cell. They observed a significant drop in methanol crossover and also noticed a high proton conductivity value [33]. In another work reported by Lee et. al., sulfonated titanium oxide (s-TiO_2_) was used as a filler with polybenzimidazole, and the performance of the composite membrane improved by 30% at 150 °C.

Among the multitude of inorganic fillers, Layered double hydroxides (LDHs), also known as hydrotalcite, have been widely investigated as inorganic fillers due to their special physicochemical properties [34,35]. LDHs, whose structure is very similar to brucite [Mg(OH)_2_], belong to a family of inorganic materials having theformula [M^2+^_1−x_M^3+^.(OH)_2_](A^n−^)_x/n_.mH_2_O, where M^2+^ represents a divalent cation such as Cu, Zn, Ni, Co, Mg, etc. while M^3+^ is a trivalent cation such as Fe, Ga, V, Al, Cr, etc., A^−^ denotes anion with charge n and x is the layer charge density [34,35]. Inorganic LDH material has metallic cation layers with a cation at the center and six oxyanions (OH^−^) attached making an octahedron [36]. Several LDH platelets are usually stacked and held together by weak interactions, typically hydrogen bonding. In their interlayer spacings there are anions (NO^−^) present which act as exchangeable ions [37,38,39,40]. As one of the key features of LDHs material as filler for PEMs, the platelets are expected to participate in the proton conduction once dispersed in the polymer matrix. Despite this, the studies reported to date are quite contrasting. Kim and coworkers reported an increase in the thermal stability and proton conductivity of the sPEEK after the introduction of LDH [41]. Similar results were obtained by Herrero et al. in the case of sPSU-based nanocomposites [35]. In contrast, Lee et al. [42,43] have demonstrated that the addition of LDH might have detrimental effects on the ion conductivity and diffusion coefficient for Nafion-based nanocomposites. Similarly, Zeng and coworkers have reported a decrease in the conductivity performance of poly(vinyl alcohol)/LDH composites compared to virgin PVA [44]. De facto, the phenomena governing the interplay between LDH materials and the hosting polymer, which might affect the physicochemical properties of the resulting nanocomposite, are still not clarified.

To fill this lack, we provide a comparative analysis with an attempt to elucidate the effect of LDHs materials on the physicochemical and ion conductive properties of three of the most used proton conductive membranes, which are Nafion, sulfonated polyether ether ketone (sPEEK) and sulfonated polysulfone (sPSU). For this study, LDH based on Mg^2+^/Al^3+^ (2:1 metals ratio) with NO_3_^−^ interlayer anion was chosen and membranes at 3 wt.% of filler loading with respect to the polymer were prepared by simple solution intercalation. According to the literature, 3 wt.% of loading has been demonstrated to be the optimal one in most of the nanocomposite systems and for various types of inorganic materials [45,46,47,48,49]. Additionally, during our previous works, we have demonstrated that the introduction of 3 wt.% of LDH material can ensure the maximum improvement in terms of transport properties, conductivity values, and mechanical properties either in the case of Nafion-based [50], sPEEK-based [51], and sPSU-based [46] composite membranes. Indeed, higher filler contents resulted in particle agglomeration, membrane inhomogeneity, and thus deterioration of the physicochemical and electrochemical performance. The microstructure of the composite membranes was investigated by X-ray diffraction, while dynamic mechanical analysis was carried out to characterize the thermomechanical performance of the various PEMs. Swelling tests and NMR spectroscopy allowed for a better comprehension of the molecular dynamics of water in the prepared membranes. In particular, the influence of the LDHs materials on the transport properties of the resulting nanocomposite membrane was investigated through direct measurements of the self-diffusion coefficients (by Pulse Field Gradient NMR method) and of the relaxation times (T_1_). Finally, the proton conductivity has been assessed by electrochemical impedance spectroscopy (EIS).

## 2. Materials and Methods

### 2.1. Materials

Nafion (20 wt.% dispersion in water and lower aliphatic alcohols) was supplied by Aldrich (Sigma-Aldrich, Milan, Italy), Polyether ether ketone (PEEK, Victrex 450PF) was purchased by ICI (London, UK) and commercial Polysulfone (Lasulf) was supplied by Lati SPA (Varese, Italy).

Sulfuric acid (95–98 wt.%), Chloroform, trimethylsilyl chlorosulfonate, sodium methoxide/methanol solution, ethanol, N,N-Dimethylformamide (DMF), N,N-Dimethylacetamide (DMAc) and Sodium Hydroxide (NaOH, 0.1 M, volumetric standard) were all purchased from Sigma-Aldrich (Sigma-Aldrich, Milan, Italy) and used as received.

### 2.2. Synthesis of Sulfonated Polyether Ether Ketone

The procedure for the synthesis of sulfonated Polyether ether ketone (sPEEK) has been deeply described elsewhere [52]. 2.5 g of polyether ether ketone (PEEK) was dried in a vacuum oven overnight at 100 °C for 24 h, then treated with 50 mL of concentrated sulfuric acid (H_2_SO_4_) and left under vigorous magnetic stirring at 25 °C till a homogeneous solution was obtained. The reaction was heated at 40 °C and left under stirring for 5 h before quenching with ice-cold distilled water. This led to precipitation of sPEEK into flake form, which was recovered by filtration and washing several times with distilled water (until pH 6–7). Finally, sPEEK was dried in a vacuum oven at 60 °C for 24 h and stored in a desiccator until use.

### 2.3. Synthesis of Sulfonated Polysulfone

Polysulfone sulfonation was achieved through the procedure described in the previous papers [13,53]. After dissolution of PSU into anhydrous chloroform at room temperature, trimethylsilyl chlorosulfonate was added to the reaction as sulfonating agent. The molar ratio between sulfonating agent and repetitive units was fixed equal to 2.5, and the reaction left at 50 °C under vigorous mechanical stirring. This allowed the production of the silyl polysulfonate derivative as an intermediate product. After 6 h, sodium methoxide solution was added dropwise to the reaction to cleave the silyl sulfonate moieties. After 1 h, polysulfone in sodium form was recovered from the solution by precipitation in a bath of ethanol, filtration, and washing several times with ethanol and distilled water. The fine powder was dried in an oven at 60 °C overnight.

### 2.4. Synthesis of Layered Double Hydroxide (LDH)

Layered double hydroxide (LDH) nanofillers were synthesized by co-precipitation in an aqueous solution of sodium hydroxide and salts of Mg^2+^ and Al^3+^, using a previously reported procedure [53]. Briefly, the magnesium/aluminum metal ratio was adjusted to 2/1 in 100 mL of an aqueous solution containing magnesium nitrate hexahydrate (0.05 mol), aluminum nitrate nonahydrate (0.025 mol), and a sodium nitrate salt (0.045 mol).

Successively, an aqueous solution of sodium hydroxide (2.5 M) was added slowly until a pH value of 10 was reached. The precipitated LDH nanomaterials were stirred at 60 °C for about 24 h and separated by centrifugation, washed several times with water, and dried at 80 °C for 24 h. The synthesis was performed under a constant flow of nitrogen and using decarbonate-deionized water. Finally, the structure of LDH was characterized using powder X-ray diffraction spectroscopy.

### 2.5. Preparation of Nanocomposite Membrane

All the nanocomposite membranes were prepared by simple solution intercalation method [51,54,55,56]. In the case of Nafion/LDH composite membrane, 1 g of commercial Nafion resin solution was heated at 60 °C until dried from the solvents and then re-dissolved in 10 mL of DMF until a clear solution was obtained. LDH was first dispersed in DMF for 48 h by alternating vigorous mechanical stirring with ultrasonication. This dispersion was then added to the solution of Nafion in DMF and left under stirring at room temperature for another 24 h. Finally, when the final solution was homogeneous, solvent-casting is performed on a Petri dish and left in the oven at 60 °C to evaporate the solvent for many hours. Pristine Nafion membrane was simply obtained from the casting of the Nafion/DMF solution. Finally, both Nafion-based membranes were subjected to thermal and chemical activations according to a method used in the literature [57].

With regard to the sPEEK/LDH and sPSU/LDH nanocomposites, an adequate amount of LDH was first dispersed in DMAc during 24 h by alternating vigorous mechanical stirring with ultrasonication. In the meantime, the selected polymer was also dissolved in 10 mL of DMAc at room temperature. Once a homogeneous dispersion was obtained, the LDH dispersions were then added to the corresponding polymer solution and left under stirring at room temperature for an additional 24 h. The membranes were obtained by casting the polymer dispersion onto a Petri dish and heating in an oven at 60 °C until dry. Pristine sPEEK and sPSU membranes were obtained by dissolving an appropriate amount of polymer into DMAc followed by casting. Before testing, the sPEEK-based [58], and sPSU-based [46,59] membranes were converted into the acid form by soaking them in a 1M H_2_SO_4_ solution for 7 h at 50–60 °C, followed by several washes with boiling deionized water to remove any residual acid.

**Figure 1:**
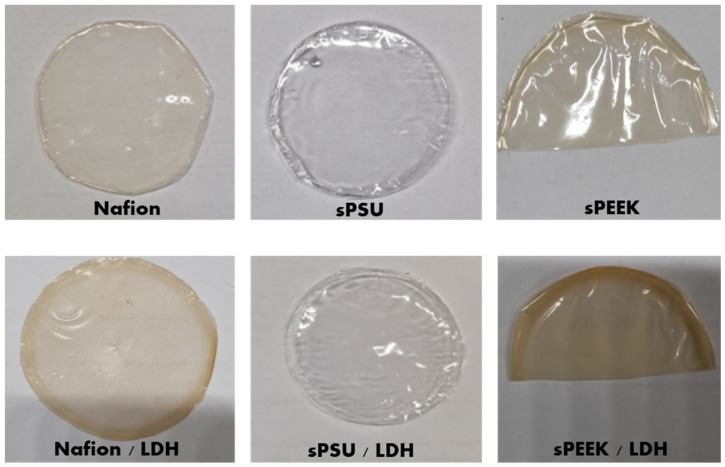
Pictures of Nafion, sPSU, and sPEEk-LDH composites (at 3% of filler loading) membranes.

For this study, all the nanocomposite membranes were prepared at 3 wt.% of loading with respect to the polymer. Figure 1 shows the photos of the membranes prepared in this study. Even after introduction of the LDH nanoplatelets inside the Nafion, sPEEK, and sPSU matrices, all the membranes still appear completely homogeneous and highly transparent. In fact, no clay particle crystals are observed suggesting the absence of agglomerates or inhomogeneity. The average thickness of the membranes ranged between 50 and 55 μm.

### 2.6. Characterization Techniques

Ion Exchange Capacity (*IEC*, in milliequivalents per g of dry polymer) for all the membranes was measured by the acid-base titration method [60]. The samples in the acid-activated form were immersed in 2M NaCl solution for 24 h at room temperature to completely release H^+^ due to the exchange with Na^+^. Thereafter, the amount of released H^+^ was titrated with standard NaOH solution (0.1 M). Phenolphthalein was used as indicator. The *IEC* values (meq g^−1^) were calculated according to Equation (1):

(1) $$IEC\;\left(\mathrm{meq}\cdot g^{-1}\right)=\frac{M_{\left(NaOH\right)}\;V_{\left(NaOH\right)}}{W_{dry}}$$

where *V* is the volume (mL) and M is the concentration (mol/L) of the NaOH solution consumed to neutralize the H+ ions, while *Wdry* is the dry weight of the sample.

Water uptake (*wu*%) was measured by soaking the dried membranes (whose weight is *w_dry_*) in deionized water at room temperature for 24 h. Each sample was then quickly dried with tissue paper to remove surface water droplets and weighted (*w_wet_*). The water uptake was calculated by Equation (2) and reported as an average of at least three independent measurements.

(2) $$wu\;\left(\mathrm{wt}.\%\right)=\frac{w_{wet}-w_{dry}}{\;w_{dry}}*100$$

Similarly, the swelling stability of the samples was evaluated in terms of dimensional variation during heating. In detail, each sample was swelled in distilled water heated at a temperature ranging from 30 to 80 °C, each 10 °C. For each temperature, equilibration time was 2 h. The water uptake was then calculated from the difference between the wet and the dry mass.

From the water content and the *IEC*, the number of water molecules per -SO_3_H group, defined as *λ* value, can be calculated by the formula reported in Equation (3):

(3) $$\lambda =\frac{wu}{MM_{H_{2}O}}\times IEC$$

X-ray diffraction (XRD) measurements were performed using the Cu-Kα radiation of a Bruker Axis Diffractometer/Reflectometer (D8) equipped with a Dynamic Scintillation Detector, NaI, and with a Göbel mirror [61]. Spectra were collected at room temperature in the 2θ range from 5° to 40°, in steps of 0.03°, and the counting time was 1 s/step [47].

NMR spectroscopy measurements were performed on a Bruker AVANCE 300 wide bore spectrometer working at 300 MHz on ^1^H [62]. The employed probe was a Diff30 Z-diffusion 30 G/cm/A multinuclear with substitutable RF inserts. The self-diffusion coefficients (D) of water confined in the membranes were measured by the pulsed field gradient stimulated-echo (PFG-STE) technique [63]. In these experiments the following experimental parameters were used: diffusion time (Δ) of 8 ms, pulse length (δ) of 0.8 ms, gradient amplitude varying from 100 to 900 G/cm and the number of scans was 8. Due to the very low standard deviation of the fitting curve and repeatability of the measurements, the uncertainties in D values were lower than 3%. Longitudinal relaxation time (T_1_) values of water were instead obtained by the inversion recovery sequence (π-τ-π/2). Measurements were conducted by increasing temperature step by step from 20 °C to 130 °C, every 20 °C, leaving the sample to equilibrate at each temperature for about 15 min.

Electrochemical Impedance Spectroscopy (EIS) was used to measure through-plane proton conductivity of all nanocomposite membranes. It used a homemade two-electrode cell connected with a fuel cell test hardware (850C, Scribner Associates, Inc., Southern Pines, NC, USA). The measurements were performed at 90% RH in the temperature range between 20 °C and 120 °C. A PGSTAT 30 potentiostat/galvanostat (Methrom Autolab) equipped with an FRA module was used to measure the AC impedance response of the cell. The AC voltage amplitude was 10 mV and the frequency ranged between 1 Hz and 1 MHz. The membrane resistance *R* was determined from the high-frequency intersection of the impedance arc with the real axis in a Nyquist plot using NOVA software 2.0. Proton conductivity, *σ* (S/cm) was calculated using the following Equation (4).

(4) $$\sigma \left(S\;cm^{-1}\right)=\frac{l}{R_{el}*A}$$

## 3. Results and Discussion

### 3.1. Morphological, Structural and Thermomechanical Properties

**Figure 2:**
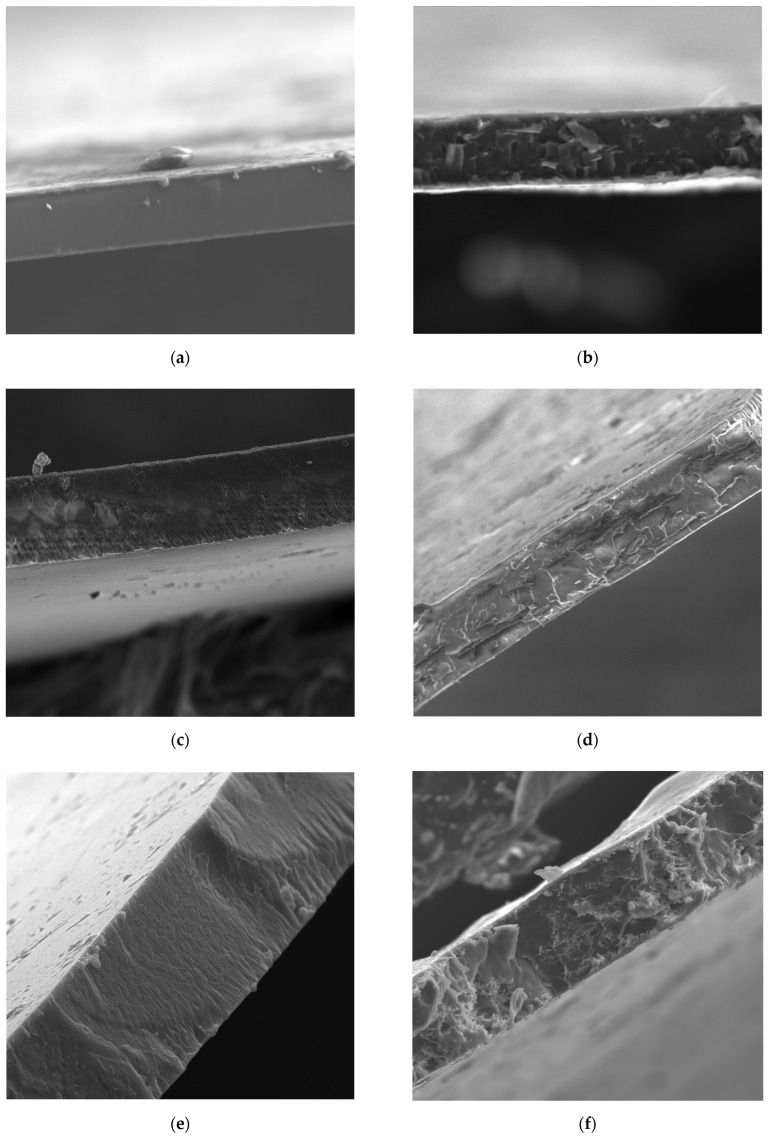
Cross-sectional SEM images of (**a**) Nafion, (**b**) Nafion/LDH, (**c**) sPEEK, (**d**) sPEEK/LDH, (**e**) sPSU, and (**f**) sPSU/LDH membranes.

Morphological characterization of Nafion-based, sPEEK-based, and sPSU-membranes was performed by Scanning Electron Microscopy (SEM), and the cross-sectional images are reported in Figure 2. It is possible to see that all the pristine membranes (Figure 2a,c,e) exhibit a dense, compact, and free-of-defect cross-section. Overall, the introduction of the LDH nanoplatelets does not significantly alter the morphological features of the PEMs (Figure 2b,d,f). However, while the plain membranes are characterized by a smooth and plain morphology, the cross-section of the composite PEMs appears more wrinkled and crumpled. Likely, the LDH platelets provide for nanosized cleavage planes during the crio-fracturing of the samples. It is worth noting that no agglomerates are visible through the cross-section of the Nafion/LDH, sPEEK/LDH, and sPSU/LDH nanocomposites. The evidence indicates that the LDH platelets are homogeneously dispersed in each hosting matrix, and thus maintain sub-micrometric dimensions.

**Figure 3:**
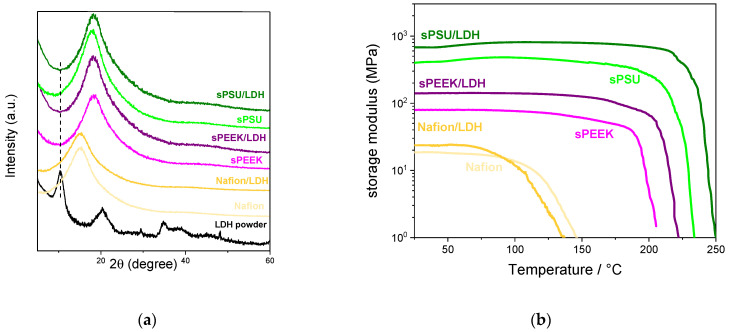
(**a**) XRD patterns and (**b**) DMA thermograms for the Nafion-based, sPEEK-based, and sPSU-based membranes.

To clarify the nature of the various composite membranes, i.e., conventional nanocomposite with stacked nanoparticles, intercalated one, or exfoliated nanocomposite, X-ray diffraction (XRD) analysis was carried out. XRD patterns of the various membranes are illustrated in Figure 3a, in comparison with that of the Mg/Al-NO_3_^−^ LDH powder. Clearly, the spectra of Nafion, sPEEK, and sPSU is characterized by the presence of a single broadband in the 2θ range of 12–30 degree, indicating all the bare membranes are completely amorphous. Worth noting, in all the nanocomposite membranes there is no evidence of the typical diffraction peaks of staked LDH platelets (which exhibit a major diffraction peak at 2θ = 10.1°), indicating the nanolamellae lost their stacking. This provides clear evidence of the fact that the solution intercalation procedure allows for obtaining completely exfoliated membranes.

Without adequate thermomechanical resistance, an electrolyte membrane could not meet the needs for membrane electrode assembly (MEA) fabrication in the fuel cell. Consequently, Dynamic Mechanical Analysis (DMA) was used to obtain crucial information about the viscoelastic properties of the investigated membranes. In this regard, Figure 3b illustrates the temperature variation of the storage modulus for the Nafion-based, sPEEK-based, and sPSU-based PEMs. The mechanical resistance increases in the order of Nafion < sPEEK < sPSU being their storage modulus 18.5 MPa, 79.1 MPa, and 390.8 MPa, respectively. It is worth noting that the sPSU membrane is able to ensure impressive mechanical strength (i.e., its storage modulus is 21-fold higher than Nafion) as well as outstanding thermal resistance. Indeed, while E′ for Nafion starts decreasing at a temperature above 80 °C, the storage modulus of sPSU remains almost stable until ca. 200 °C. The outcome holds promise for the successful utilization of sPSU under PEM-FCs operating under very high temperatures. Following the introduction of the LDH nanoplatelets, the storage modulus increases with respect to the virgin polymers, no matter the hosting matrix. However, while the enhancement is quite moderate in the case of Nafion/LDH, it became significant in the case of sPEEK/LDH and sPSU/LDH. Noticeably, E′ increases to a similar extent for the two nanocomposite membranes based on polyaromatic polymers: storage modulus of the sPEEK/LDH and sPSU/LDH membranes is almost 80% higher with respect to the bare polymers. Such an impressive increase in membrane resistance is quite typical for nanocomposite membranes comprising 2D-layered materials [48,64,65]. The outcome can be ascribed to the inherent capacity of this class of fillers to generate a nacre-like structure [66]. Indeed, the anionic clays experience strong electrostatic interaction with the functional groups of the hosting matrix but also with the platelets themselves. Due to this synergistic effect, the mechanical stress can be rapidly transferred from the polymer matrix to the high-strength LDH nanoparticles in a manner similar to nacre [58,67]. Last but not least, the mutual interaction between LDH platelets and polymer backbone also provides for better thermal resistance, thus further extending the operating temperature of the PEMs. Indeed, the decrease in the storage moduli for nanocomposite membranes based on sPEEK and sPSU is shifted at higher temperatures compared to the virgin membranes.

### 3.2. Ion Exchange Capacity and Water Uptake Behavior

The ion exchange capacity (IEC, meq g^−1^) of a membrane relates to the number of exchangeable ions contained in a membrane, consequently, it gives an estimation of the number of functional groups available for proton conduction [46]. The IEC values for the various Nafion-, sPEEK- and sPSU-based membranes are summarised in Table 1 together with the water uptake at 20 °C. Comparatively, all the nanocomposite membranes exhibit higher IEC values than the parental polymers. This can be clearly ascribed to the charged nature of the anionic lamellae dispersed in the polymer matrices, which produces an appreciable increase in the number of polar groups. The highest IEC value of 2.13 meq g^−1^ was achieved by the sPEEK/LDH membrane. The variation in IEC typically impacts the water absorption capacity of the electrolyte: generally, the higher the IEC, the higher the hydrophilicity and thus, the higher the water uptake. This is valid for the Nafion-based PEMs, since the w.u. increases from ca. 25 wt.% in the bare polymer to 28.3% in the Nafion/LDH. Contrariwise, the introduction of the LDH lamellae inside both polyaromatic polymers produces a slight decrease in the maximum absorption capacity. This peculiar outcome strongly suggests that the nanoclay impacts the microstructure of the hydrophilic channels of both nanocomposites. Due to their positively charged surface, the LDH platelets may act as physical crosslinkers between -SOH functional groups of adjacent polymers chains, decreasing the overall free volume in sPSEEK/LDH and sPSU/LDH and thus limiting their swelling capability.

**Figure 4:**
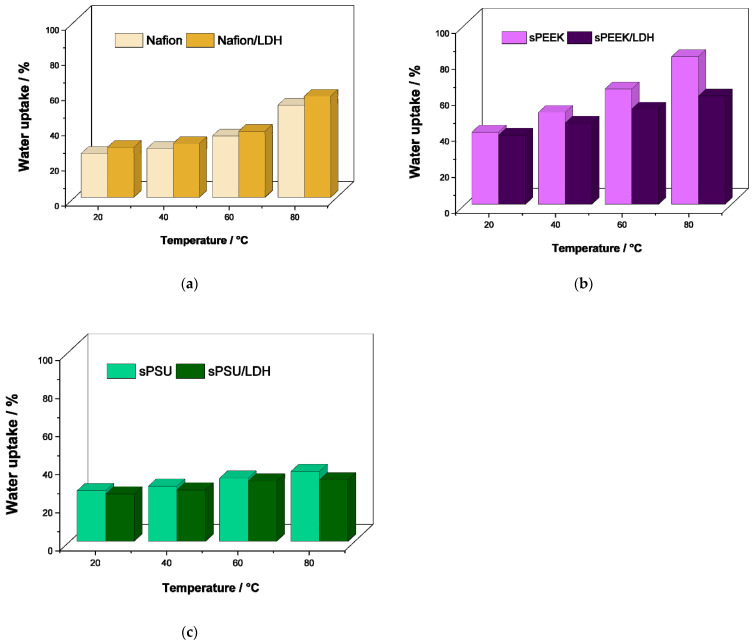
Temperature evolution of water uptake for (**a**) Nafion-based, (**b**) sPEEK-based and (**c**) sPSU-based membranes.

To clarify the effect of the LDH introduction on the hydrolytic resistance of the various nanocomposite membranes, their swelling behavior was investigated under variable temperatures. In this regard, Figure 4 shows the temperature evolution of the water uptake for the Nafion-based (Figure 3a), sPEEK-based (Figure 4b), and sPSU-based (Figure 4c) membranes, in the temperature range 20–80 °C. Both Nafion and sPEEK membranes exhibit a massive swelling during heating, with an increase of more than 50% in the water uptake at 80 °C. This is predictive of low dimensional stability which typically leads to deterioration of mechanical resistance and, most importantly, to dimensional mismatch when the membrane is assembled into a fuel cell [68]. Obviously, the highest swelling is reached by bare sPEEK due to the very high IEC value. In the case of bare sPSU, the swelling tests revealed the membrane has an excellent anti-swelling capability: its water uptake increases from ca. 27 wt.% at 20 °C to 37 wt.% at 80 °C. Turning the attention to the nanocomposite membranes, it can be clearly seen that the introduction of the LDH poorly impacts the swelling features of Nafion. De facto, the filler particles simply increase the number of hydrophilic sites in the PEM. On the other side, the anionic clays have a beneficial impact on the dimensional stability of both sPEEK and sPSU due to the massive reduction in swelling during heating. Such an outcome is very positive because it guarantees the dimensional stability of the electrolyte membrane during the PEMFC operation.

**Figure 5:**
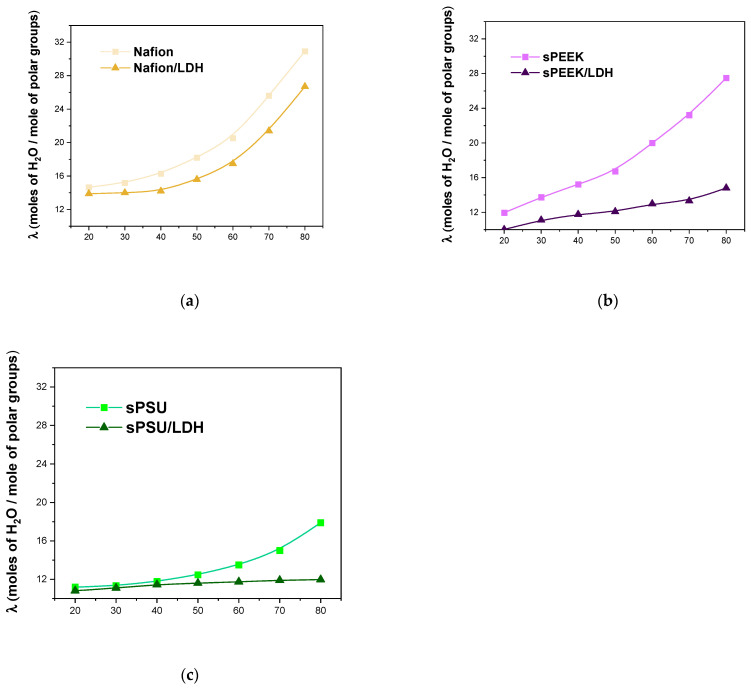
Temperature evolution of λ for (**a**) Nafion-based, (**b**) sPEEK-based, and (**c**) sPSU-based membranes.

This is further corroborated by the temperature evolution of the lambda (λ) or coordination number, described as the number of water molecules per sulfonic acid group. The λ values provide crucial information concerning the microstructure of the electrolyte films. The values for the investigated membranes, for the temperature range 20–80 °C, are illustrated in Figure 5. The λ value increases (massively for Nafion and sPEEK whereas moderately for sPSU) with the temperature due to the softening of the polymer chains under the thermal energy, which can thus accommodate a larger amount of water. It is worth noting that, for all the hosting matrices, the λ value declined remarkably after the addition of LDH. As discussed above, the nanocomposite membranes exhibit higher IEC but similar water uptake compared to the parental polymers. This means in the membranes containing the LDH nanoplatelets there is a larger number of hydrophilic groups but a lower number of water molecules solvating them. Moreover, while the addition of LDH particles inside the Nafion matrix does not impact the dimensional variation of the ionic clusters with the temperature, the number of water molecules per sulfonic acid group barely increased during heating for both sPEEK and sPSU nanocomposites. This definitely confirms that in these polymers the introduction of the LDH minimizes the alteration of the cluster size during heating due to the aforementioned crosslinking activity of the LDH lamellae.

### 3.3. ^1^H Nuclear Magnetic Resonance (D and T_1_) Characterization

**Figure 6:**
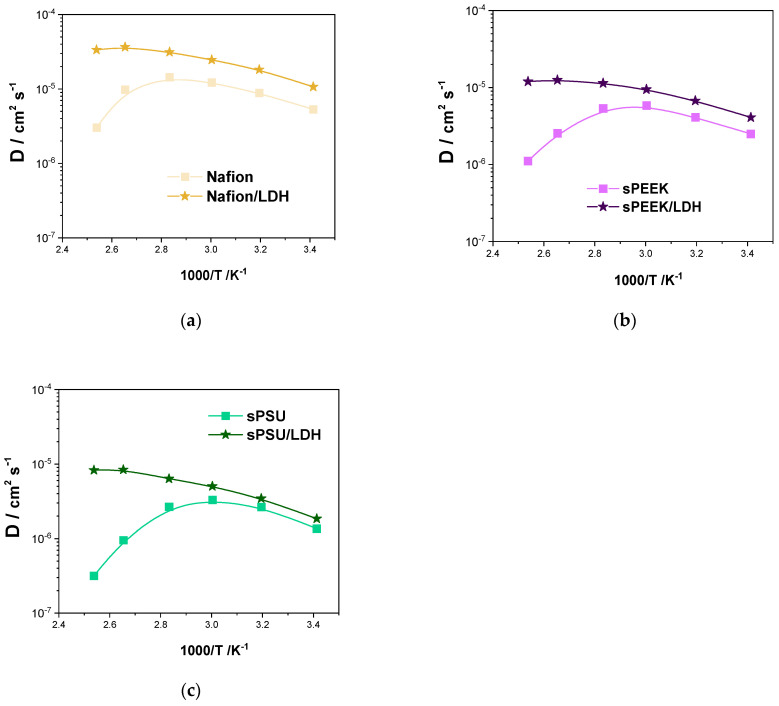
Self-diffusion coefficients and as a function of the temperature (from 20 °C to 120 °C) of the water confined in (**a**) Nafion and Nafion/LDH, (**b**) sPEEK and sPEEK/LDH and (**c**) sPSU and sPSU/LDH.

^1^H-NMR spectroscopy allowed study of the molecular dynamics (both long- and short-range) of water confined inside the hydrophilic clusters of the prepared nanocomposite membranes, through the direct measurements of the water self-diffusion coefficients (D) and the longitudinal relaxation times (T_1_), respectively. Figure 6 shows the water self-diffusion coefficients measured on completely swollen membranes (that is, at the maximum water uptake) in the temperature range of 20–120 °C. Typically, water diffusivity in PEMs increases with the temperature due to thermal energy, but then abruptly decreases once the evaporation of mobile bulk water becomes massive. The crucial temperature for bare Nafion is 100 °C, while it is shifted toward a lower temperature (80 °C) in the case of sPEEK and sPSU. Above these temperatures, only water molecules solvating the acid groups of the polymers contribute to D, but they are almost immobile. This is the reason for such a drop in diffusivity, which de facto limits the performance of the corresponding PEM-FCs operating under high temperatures. It can be clearly seen that the presence of the LDH nanoplatelets improves the diffusivity of the resulting nanocomposite, no matter the hosting matrix. Noteworthy, the beneficial impact of the filler becomes more appreciable in the high-temperature region, i.e., above 80 °C. Indeed, D keeps on increasing for all the nanocomposites till 120 °C, indicating the anionic platelets are able to retain a satisfactory amount of mobile water even under a dehydrating environment. Comparatively, the best performance was achieved by the sPSU/LDH membrane, which exhibits a self-diffusion coefficient of ca. 1 × 10^−5^ cm^2^ s^−1^ at 120 °C which is almost two orders of magnitude higher than the parental polymer. This holds promise for the successful implementation of this kind of nanocomposite membrane into high-temperature PEM-FCs.

**Figure 7:**
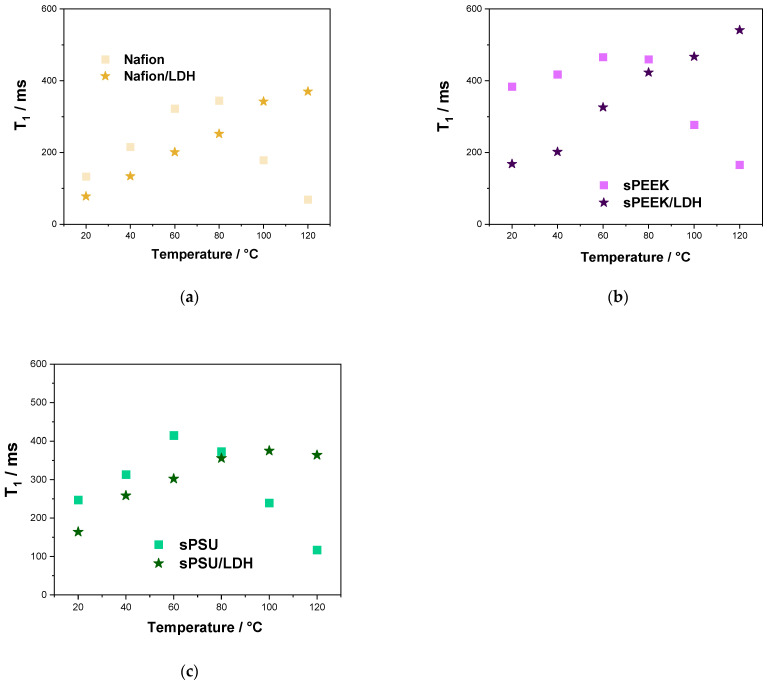
T_1_-relaxation times as a function of the temperature (from 20 °C to 120 °C) of the water confined in (**a**) Nafion-based, (**b**) sPEEK-based and (**c**) sPSU-based membranes.

The analysis of spin-lattice relaxation times (T_1_) was able to shed light on molecular interactions of water molecules inside the investigated membranes. Compared to D, T_1_ refers to more localized motions including both translation and rotation on a time scale comparable to the reciprocal of the NMR angular frequency (a few nanoseconds). In a nutshell, the larger the interactions between spin and lattice, the quicker the relaxation (shorter T_1_), with T_1_ generally increasing with the temperature [69]. Figure 7 reports the temperature behavior of T_1_ in the range of 20–120 °C. We can observe that T_1_ values decrease for all the polymer matrices after the addition of the LDH lamellae, confirming the filler provides for stronger dipolar interactions, and faster decays of longitudinal magnetization (shorter T_1_), with the water molecules. Since the Grotthuss mechanism relies on the creation of a highly-interconnected path for ion transport, stronger interactions between the H^+^ ions and the lattice generally provides for higher proton conductivity [70].

### 3.4. Proton Conductivity

**Figure 8:**
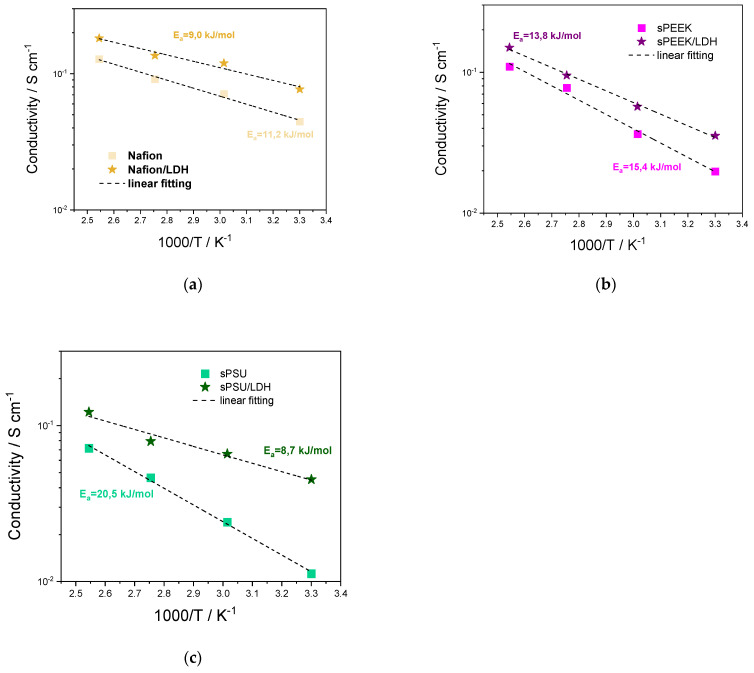
Arrhenius plots of proton conductivities at 90% RH of (**a**) Nafion and Nafion/LDH, (**b**) sPEEK and sPEEK/LDH and (**c**) sPSU and sPSU/LDH. Dash lines represent the linear fitting to experimental data.

The Arrhenius plot of the proton conductivity (σ) measured on pristine and nanocomposite membranes is shown in Figure 8 at 90% relative humidity conditions in the temperature range of 30–120 °C. Some representative values are also reported in Table 2. By comparing the bare polymers, it is clear that Nafion exhibits superior performance in terms of proton conductivity in spite of its very low IEC (i.e., 0.93 meq g^−1^). The membrane possesses a peculiar microstructure where the hydrophobic PTFE backbone is well separated by the hydrophilic ion conducting clusters [71,72]. This clear phase segregation provides for very high proton conductivity, at least under highly-humidified conditions: the bare Nafion is able to yield 128 mS cm^−1^ at 120 °C. Even if less pronounced, a sort of nanoscale segregation can be hypothesised also for the sPEEK membrane, but the ionic clusters are narrower and poorly interconnected resulting in lower σ values with respect to Nafion [73,74]. Contrariwise, sPSU lacks any phase separation, leading to the formation of lamellar, very narrow, and highly branched hydrophilic clusters with a larger number of dead-end “pockets” [75]. This is clearly corroborated by the Activation Energy (Ea) calculated for the proton conductivity in the three polymers, which increases from 11.2 kJ mol^−1^ in the case of Nafion, to 15.4 kJ mol^−1^ for sPEEK. sPSU exhibits the lowest conductivity as well as the highest Ea, i.e., 20.5 kJ mol^−1^, confirming the very low efficiency of the proton transport in this membrane.

The appropriate nanodispersion of the anionic nanoclay boosts the proton conduction, independently from the hosting matrix. Indeed, the LDH platelets provide for additional sites that are directly involved in the proton conduction via the Grotthuss mechanism, as confirmed by the impressive reduction in the Ea following the introduction of the nanofiller. Quite surprisingly, the major improvement is observed in the case of the qPSU/LDH membrane which reaches a σ of 122 mS cm^−1^ at 120 °C, almost attaining the performance of the Nafion, which is the current benchmark for the application in PEM-FCs. The sPSU/LDH membrane also shows the lowest activation energy (8.7 kJ mol^−1^) among the investigated membranes. As mentioned above, acting as a physical crosslinker, the LDH nanoplatelets are able to fill the gap between adjacent sulfonic acid groups, thus enabling highly-efficient proton conduction even in sPSU.

**Figure 9:**
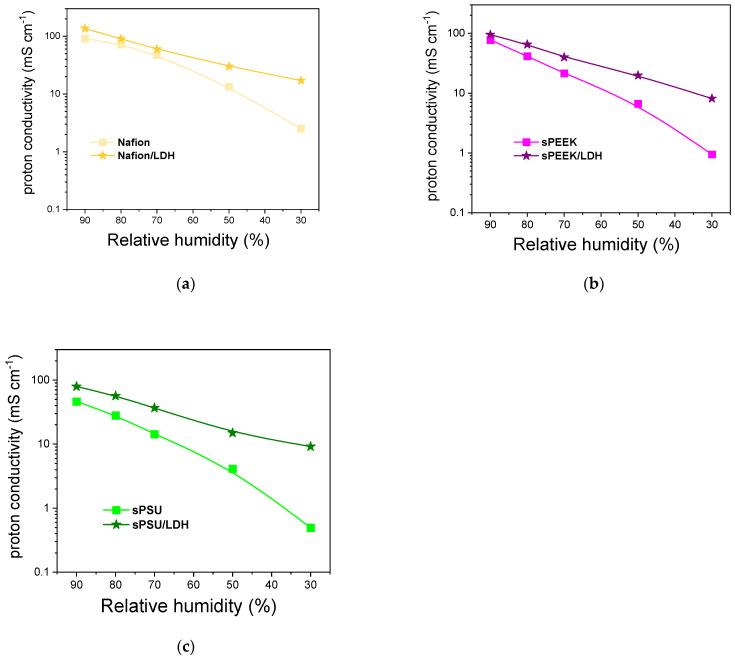
Proton conductivity vs. relative humidity at 90 °C for (**a**) Nafion and Nafion/LDH, (**b**) sPEEK and sPEEK/LDH, and (**c**) sPSU and sPSU/LDH membranes.

Finally, proton conductivity was also investigated at 90 °C and under different RH conditions (from 30 to 90% RH). The results are shown in Figure 9. As expected, pristine membranes exhibit a significant drop in conductivity as the relative humidity decreases from 90% to 30%. In the “quasi-anhydrous” state, the performance of Nafion decreases by one order of magnitude, while the drop in conductivity is even higher for both sPEEK and sPSU: for these two polymers, σ at 30% RH is more than two orders of magnitude lower than that achieved at 90% RH. It is worth noting that, as the booster of the proton conductivity, the LDH plays a major role under low humidification conditions, where progressive depletion of water molecules is partially compensated by the presence of the anionic lamellae which are directly involved in the proton transport mechanism. Both sPEEK/LDH and sPSU/LDH membranes yield a proton conductivity of ca. 10 mS cm^−1^ @ 90 °C and 30% RH, significantly exceeding the performance of Nafion. In a nutshell, the introduction of LDH could be exploited to convert cost-effective but low-performing polyaromatic polymers into PEMs able to ensure satisfactory proton conductivity even under dehydrating conditions. The latter is a very important requirement for the large-scale development of high-temperature PEM-FCs.

## 4. Conclusions

The preparation of nanocomposite membranes seems to represent, to date, the most effective path to overcome the performance, safety, and cost limitation of conventional perfluorosulfonic acid (PFSA) polymer for PEM-FCs applications. Among the multitude of fillers, layered double hydroxides (LDHs) have been emerging as very promising materials due to their peculiar physicochemical and electrochemical properties.

Herein, several nanocomposite membranes were prepared by LDHs nanoparticles (Mg^2+^/Al^3+^ LDH with a metal ratio of 2:1 and NO_3_^−^ interlayer anions) in three different polymer matrices: one perfluorosulfonic acid polymer, i.e., Nafion, and two polyaromatic polymers such as sulfonated polyether ether ketone (sPEEK) and sulfonated polysulfone (sPSU). The filler loading was kept at 3 wt.% for all the nanocomposite membranes. To elucidate the effect of the filler introduction on the physical-chemical and electrochemical properties of the nanocomposite membranes, the PEMs were then characterized in terms of microstructure, thermo-mechanical resistance, proton mobility, and electrochemical performance. XRD revealed the solution intercalation procedure which allowed the production of completely exfoliated membranes, that is, the nanoplatelets lose their staking, no matter the hosting matrix. Under this circumstance, the anionic lamellae are able to massively enhance the thermomechanical resistance (i.e., to 80% improvement of the storage modulus) of the resulting membrane likely due to the formation of a nacre-like structure. Both swelling tests and ^1^H NMR characterization confirmed that in the case of Nafion-based membranes, the LDHs simply provide for additional hydrophilic sites that increase both water adsorption and water retention capacity. Contrariwise, the introduction of LDH into sPEEK and sPSU perhaps alters the microstructure of the ionic clusters leading to improved proton transport properties but also to impressive anti-swelling capability. De facto, the anionic clays act as a physical crosslinker between adjacent sulfonic acid groups, both in sPEEK and sPSU. This also promotes the formation of highly-conductive paths for proton transport that dramatically enhance the conductivity performance of the two nanocomposites. Noteworthy, sPSU/LDH nanocomposite exhibited a peak conductivity performance of 122 mS cm^−1^ at 120 °C and 90% RH, almost attaining the performance of the Nafion, i.e., the current benchmark for the application in PEM-FCs. The results of this study demonstrated that the nature of the interaction between the filler and the hosting matrix plays a crucial role in determining the overall performance of the final composite membrane. Additionally, due to the low-cost, ease of preparation, and eco-friendly nature of the sPSU/LDH nanocomposite, it has tremendous potential as an alternative polymer for large-scale application in PEM-FCs.

## Figures and Tables

**Table 1:** Ion Exchange Capacity (IEC) and water uptake (W.U.) values for the various pristine and nanocomposite membranes.

Membrane	IEC [meq g^−1^]	W.U. @ 20 °C [wt.%]
Nafion	0.93	24.8
Nafion/LDH	1.19	28.3
sPEEK	1.91	40.0
sPEEK/LDH	2.13	38.4
sPSU	1.39	26.5
sPSU/LDH	1.49	24.8

**Table 2:** Conductivity values at two representative temperatures (i.e., 30 and 120 °C) and humidification conditions (30 and 90% RH) for the various PEMs.

Membrane	σ at 30 °C [mS cm^−1^]	σ at 90 °C [mS cm^−1^]		σ at 120 °C [mS cm^−1^]
	90% RH	30% RH	90% RH	90% RH
Nafion	44.52	2.51	91.08	127.91
Nafion/LDH	76.90	17.10	136.04	182.07
sPEEK	19.78	0.95	77.56	109.72
sPEEK/LDH	35.47	8.14	95.04	149.59
sPSU	11.21	0.49	46.3	71.60
sPSU/LDH	45.22	9.31	79.3	128.25

## Data Availability

Not applicable.
