# Correction: Rehman et al. Nanocomposite Membranes for PEM-FCs: Effect of LDH Introduction on the Physic-Chemical Performance of Various Polymer Matrices. *Polymers* 2023, *15*, 502

**DOI:** 10.3390/polym16182673

**Published:** 2024-09-23

**Authors:** Muhammad Habib Ur Rehman, Ernestino Lufrano, Cataldo Simari

**Affiliations:** 1 Department of Chemistry and Chemical Technologies, University of Calabria, 87036 Rende, Italy; 2 National Reference Centre for Electrochemical Energy Storage (GISEL)—INSTM, Via G. Giusti 9, 50121 Firenze, Italy

**Error in Figure**

In the original publication [1], the authors identified a mistake in Figure 3a as published. The raw data for the XRD spectra of sPSU/LDH membrane were reported twice as sPSU/LDH and sPEEK/LDH. The corrected Figure 3a appears below. The authors state that the scientific conclusions are unaffected. This correction was approved by the Academic Editor. The original publication has also been updated.

## Figures and Tables

**Figure 3:**
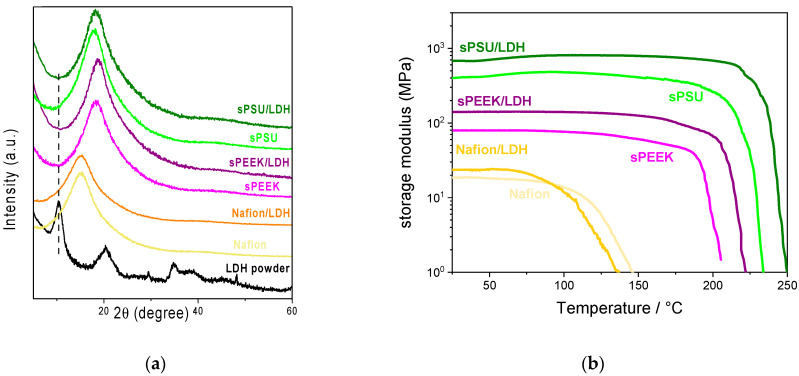
(**a**) XRD patterns and (**b**) DMA thermograms for the Nafion-based, sPEEK-based, and sPSU-based membranes.
